# Child Care in Times of COVID-19: Predictors of Distress in Dutch Children and Parents When Re-entering Center-Based Child Care After a 2-Month Lockdown

**DOI:** 10.3389/fpsyg.2021.718898

**Published:** 2021-11-05

**Authors:** Sanne M. de Vet, Claudia I. Vrijhof, Shelley M. C. van der Veek, Jane M. Pieplenbosch, Hedwig J. A. van Bakel, Harriet J. Vermeer

**Affiliations:** ^1^Parenting, Child Care and Development, Faculty of Social and Behavioral Sciences, Institute of Education and Child Studies, Leiden University, Leiden, Netherlands; ^2^Department of Tranzo, Tilburg School of Social and Behavioral Sciences, Tilburg University, Tilburg, Netherlands

**Keywords:** re-entering center-based child care, child and parental distress, COVID-19, early childhood, parental anxiety

## Abstract

As a consequence of the outbreak of the Coronavirus Disease 2019 (COVID-19) child care facilities all over the world were temporarily closed to minimize the spread of the virus. In Netherlands, the first closure lasted for almost 2 months. The return to the child care center after this significant interruption was expected to be challenging, because earlier studies demonstrated that transitions into child care can be stressful for both children and their parents. The current paper retrospectively examined the distress of Dutch children (aged 0–4) and their parents during the first 2 weeks after the reopening of child care centers, and what factors accounted for individual differences in distress. In total, 694 parents filled out an online questionnaire about stress during closure and distress after the reopening of child care centers. Furthermore, questions regarding several demographic variables and child care characteristics were included, as well as questionnaires measuring child temperament, parental separation anxiety, and parental perception of the child care quality. Results showed that younger children and children with parents scoring higher on separation anxiety experienced more distress after the reopening, as reported by parents. Furthermore, children were more distressed upon return when they attended the child care center for less hours per week after the reopening, experienced less stress during closure, and grew up in a one-parent family. With regard to parental distress after the reopening, we found that parents scoring higher on separation anxiety and fear of COVID-19 experienced more distress. Moreover, parents experiencing less stress during closure and mothers were more distressed when the child returned to the child care center. Finally, concurrent child and parental distress after reopening were positively related. The results of the current study may help professional caregivers to identify which children and parents benefit from extra support when children return to the child care center after an interruption. Especially the role that parental separation anxiety played in predicting both child and parental distress deserves attention. More research is required in order to study the underlying mechanisms of these associations and to design appropriate interventions.

## Introduction

The outbreak of COVID-19 and the accompanying lockdowns have had an enormous impact on societies and individuals worldwide. It not only caused an immediate international health crisis, but it also gave rise to different challenges regarding other aspects of daily life. The closure of schools and child care centers during lockdowns have put a large strain on families with young children, as has been studied considerably (e.g., Brown et al., 2020; Del Boca et al., 2020; Jones, 2020; Russell et al., 2020; Huebener et al., 2021). However, the return to normal life after the withdrawal of measures deserves attention too. We know from earlier studies that transitions into child care can be stressful for both children and their parents (e.g., Ahnert et al., 2004; Cryer et al., 2005; Klein et al., 2010; Swartz et al., 2016), and returning to the child care center after a 2-month interruption (which was the case in Netherlands during the first national lockdown) might therefore have been challenging for children and parents as well. This idea is supported by a study from the United States (Jones, 2020) that showed that around 85% of parents and professional caregivers expressed their concerns regarding the reopening of child care centers. Therefore, in the current study, the distress as experienced by parents and children (as reported by parents) upon children’s return to the child care center was examined. More knowledge on predictors of distress could guide policy makers in comparable future situations and may help professional caregivers to identify children and parents who are most in need of extra support when children re-enter the child care center after a long period of absence.

### Transitions Into Child Care

Earlier studies pointed out that transitions into new child care settings can cause distress for children, resulting in higher cortisol secretion at child care compared to home (Bernard et al., 2015; Albers et al., 2016), especially during the first 2 weeks after the start at the child care center (Ahnert et al., 2004). Furthermore, infants and toddlers showed more behavioral discontent, as indicated by more crying, fussing and clinging to caregivers during the first month after transitioning into a new child care group (Cryer et al., 2005). These observations are in line with attachment theory, which has shown in abundance how trying separations from primary attachment figures can be for (young) children (e.g., Klette and Killén, 2019). Less is known about parental distress during transitions. The small number of studies that do exist show that some parents also experience distress when their child transitions into an out-of-home child care setting (Klein et al., 2010; Swartz et al., 2016). For example, in the study by Swartz et al. (2016) on maternal perspectives regarding the transition of their child into child care, it was found that 39.7% of the mothers were classified as experiencing the transition of their child as difficult themselves.

The process of adjusting to the child care setting after a significant interruption such as after the lockdown, is likely to resemble adjustment to a new child care setting. Below, we describe what is known about child and parental factors in relation to the adjustment to a (new) child care setting. It is conceivable that these factors are also important in explaining individual differences in child and parent distress when children and parents re-adjust to the child care center after an interruption. Therefore, we investigated these factors in the current study. Furthermore, several COVID-19 related factors that might have played a role in the reactions of children and parents after the reopening will be discussed and examined.

### Child-Related Predictors

First, with regard to child characteristics, we know that children’s temperament can affect how they adapt to child care (Crockenberg, 2003; De Schipper et al., 2004). It has been shown that children scoring high on fearfulness and irritability have more difficulty adjusting to a child care setting, reflected by higher cortisol levels (Groeneveld et al., 2010), lower well-being (De Schipper et al., 2004), and behavioral difficulties during separations (Swartz et al., 2016). However, a direct link between negative affectivity and stress at child care was not always found (e.g., Albers et al., 2016). Besides negative affectivity, the degree of extraversion of children might also be related to their adjustment to a new child care setting. The transition into child care might be easier for more outgoing children, because they may be less overwhelmed by the new faces and environment, and make contact with the professional caregivers and other children more easily than introverted children. Child temperament might also affect parental distress after reopening, because parents of children with a more difficult temperament might expect a less smooth transition for their child. This could result in more stress (Östberg and Hagekull, 2000) and more negative parental emotions regarding the start of their child at the child care center.

Another relevant child level factor may be the number of hours that children spend in child care. It has been reported that more hours in child care are (moderately) associated with more negative child outcomes, such as behavioral problems (National Institute of Child Health and Human Development Early Child Care Research Network, 2003) and higher stress levels (Lumian et al., 2016). However, for the current study, which focuses on re-adjustment to the child care setting after a 2-month interruption, the number of hours might relate inversely. It could be that especially the children who are attending the child care center for only 1 day a week have more difficulty to adjust, because adjustment takes time for all parties involved. Therefore, the same might apply to the parents of these children, who also need time to get used to their child re-entering the child care center and this might be easier for parents when children attend the child care center for more than 1 day a week.

The final child characteristic this study focuses on that might explain differences in distress around transitions into child care is children’s age. Regarding child age, results are mixed, with studies finding younger children to experience more distress during transitions (Fein et al., 1993; Cryer et al., 2005) and studies showing more distress in children beyond infancy (e.g., Swartz et al., 2016). The first might be explained by younger children having less self-regulatory capacities, while Swartz et al. (2016) suggested that older children have developed stronger attachments to parents and therefore might be more wary of (relatively) unfamiliar caregivers at child care. Mothers described transitions with younger children as easier for themselves, possibly because they believed their child to be unaware of the transition (Swartz et al., 2016), which illustrates the possible link between child age and parental distress.

### Parent-Related Predictors

An important parental factor that might explain differences in child and parental distress during the transition into a child care setting is parental separation anxiety, which can be described as “a parent’s experience of worry, sadness, or guilt during short-term separations from the child” (Hock and Schirtzinger, 1992, p. 93). Although separation anxiety has been studied more extensively in mothers, fathers may experience separation distress to a similar extent (Kirby et al., 1994). It is likely that general parental separation anxiety in the context of child care but not related to a specific moment, influences how parents react emotionally to the specific situation of the (re)start of their child into the child care setting. Children of parents who experience more separation anxiety might be in turn unconsciously influenced by these feelings or experience distress because of certain unhelpful parenting practices that arise from parents’ separation anxiety. In a study of Israeli-Druze families, maternal separation anxiety was associated with more child separation distress and poorer child adjustment to the child care center (Peleg et al., 2006). Furthermore, maternal distress in response to child distress, a proxy for parental separation anxiety, was found to be associated with less smooth transitions for both children and mothers (Swartz et al., 2016).

Another parental factor is child care quality as perceived by the parent. It was found that parental perceptions of the quality of child care were associated with parental stress (Bigras et al., 2012): parents who thought the child care center of their child was of high quality experienced less stress. For child distress, mothers indicated that more support of the professional caregiver toward the child, which can be seen as an indicator of child care quality, was related to an easier transition for children (Swartz et al., 2016). This support can help children with co-regulating their emotions when they are confronted with the transition, which could explain the easier transition for children in case of more support.

### COVID-19-Related Predictors

Several child and parental factors that are more directly related to the pandemic could also be associated with child and parental distress after the reopening of child care centers, such as parental fear of COVID-19 and child and parental stress during the closure of the child care centers. One study found that throughout the 2009 Swine Flu in Netherlands, parental fear of the disease predicted child fear, partly *via* the transmission of threat information (Remmerswaal and Muris, 2010). For parents, fear of COVID-19 might have influenced how they felt about the return of their child to the child care center, as parents and children were more exposed to health risks as they left their homes. Therefore, parental fear of COVID-19 might predict parental distress after the reopening of child care centers directly and child distress indirectly.

It is likely that during the closure of child care centers, parents experienced stress because they had to combine work (at home) with the care of their child(ren), while children might have suffered from the disruption of normal routine and contacts (e.g., Orgilés et al., 2020). It could be that higher stress levels during the lockdown for both children and parents are related to higher stress levels after reopening, because children and parents who experience more stress during one challenging situation might also experience more stress during another, due to their circumstances or personal characteristics. However, children who experienced more stress because they missed the child care center to a larger extent were perhaps more excited to start again. Furthermore, parents who experienced higher stress levels during the lockdown might have been relieved that they could bring their child to the child care center after 2 months. How child and parental stress during closure could be related to emotional responses after the reopening is therefore difficult to predict, as both directions seem plausible.

### Concurrent Child and Parental Distress

Finally, the effect of parental distress after the reopening of child care centers on concurrent child distress and vice versa was examined in the current study. It is quite well-established that parental emotional reactions co-determine how children cope throughout and after disruptive events. For example, Wilson et al. (2010) found that parental reactions regarding the 9/11 terrorist attacks predicted children’s post-traumatic stress symptoms after indirect exposure. Another study showed a positive relation between the intensity of parental distress at the time of an accident and subsequent child trauma symptomatology 5–8 weeks after the event (Gallo et al., 2019). A reciprocal process in which children also influence how parents cope has been proposed as well, although child functioning was found to predict parental outcomes in a smaller number of studies (Cobham et al., 2016). More specifically related to the COVID-19 crisis, Chartier et al. (2021) found a significant association between parental and child traumatic stress related to the lockdown measures. All these studies make clear that parental and child distress around disruptive events are likely to influence each other.

### Aims of the Study

In sum, the objectives of the current study were to investigate whether Dutch children aged 0–4 years and their primary caregiver experienced distress in the first 2 weeks following their return to the child care center after a 2-month lockdown (according to the parent), and what factors accounted for individual differences in child and parental distress. With regard to child characteristics, we expected that children scoring higher on negative affectivity and lower on extraversion would be more distressed upon return, and we expected their parents to feel more distressed as well. Regarding parental factors, higher parental fear of COVID-19, higher parental separation anxiety, and lower child care quality as perceived by the parent were expected to be related to more distress in both children and parents after reopening. Regarding the other predictors, no specific hypotheses were formulated, because of the exploratory nature of these factors (hours in child care, and child and parental stress during closure) or inconclusive findings in earlier studies (child age). Finally, we expected parental and child distress after the reopening of child care centers to be related positively.

## Materials and Methods

### Procedure

The current study was approved by the Ethics Committee of the Institute of Education and Child Studies of Leiden University (ECPW-2020/283). From the 7th of August until the 7th of September 2020 (12.5–17 weeks after the official reopening of child care centers), an anonymous survey was administered *via* Qualtrics Survey Software. The recruitment text with a short summary of the study and the link to the questionnaire was placed on the website of the University, on social media, and distributed online with the help of child care organizations that participated in or showed interest for an earlier research project on child care. Different branch organizations, journals for practice and interest groups helped with the distribution as well, to try to gain national coverage. The introductory section of the survey contained detailed information about the study and questionnaire, and a question to ensure the inclusion criteria were met, i.e., the age of the child was between 0 and 4 years at the time of the reopening of the child care centers, the child had started at a regular center-based child care center before the closure, and the child resumed care after the reopening for at least 2 weeks at the same child care group. The reason for these inclusion criteria was that we wanted to exclude child reactions due to a normal adjustment process when starting at child care or a new group. A second question was inserted to make sure that the parent who filled out the questionnaire was the parent who most frequently brought the child to the child care center during the first 2 weeks after the reopening (because this parent had the most firsthand memories). When parents brought their child to the child care center equally often, they were free to choose who would fill out the questionnaire. We specifically stated that parents could discuss the questions on child reactions upon return with each other. If parents were part of the target group of the study, they were provided with the informed consent. When parents had more than one child, they were asked to fill out the questionnaire for their youngest child that met the inclusion criteria. The questionnaire took around 20–30 min to fill out, but pausing and continuing later was possible.

As an incentive, €20 gift cards to spend on toys were distributed to five randomly selected parents who completed the entire questionnaire and indicated they wanted to join the lottery. Participants could also indicate whether they wanted to receive a report on the most important outcomes of the research project in due time. Both joining the lottery and receiving a report required participants to share their e-mail address with us, which was collected through a separate questionnaire to avoid linkage between their answers and personal data. Since the questionnaire might have elicited negative emotions, we added information about several organizations at the end that parents could reach out to in case they needed support.

### Participants

**FIGURE 1:**
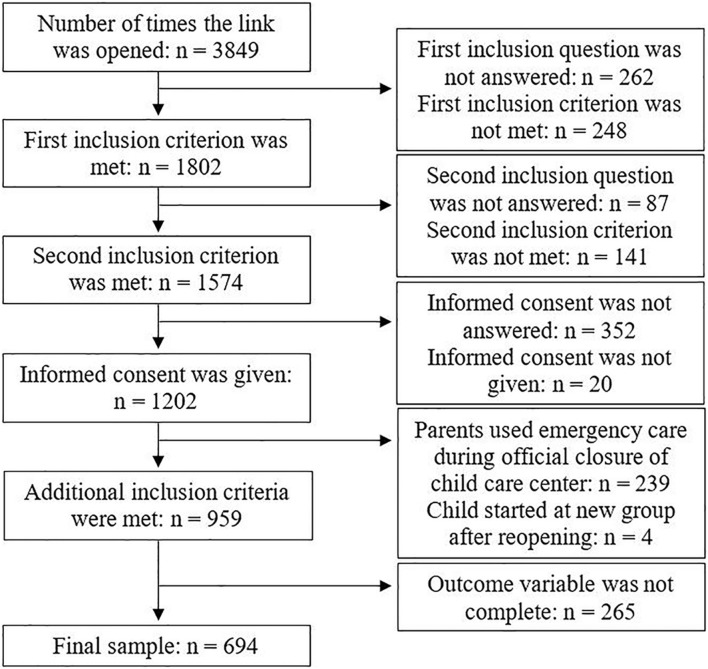
Flow chart of the sample selection process.

In Figure 1, a flowchart of the selection process for the final sample is displayed. Parents who brought their child to the child care center for emergency child care during the official closure of child care centers—because of their vital profession—were excluded from the current sample, because the situation of these participants was not comparable to that of the other participants. For four participants, it appeared from their answers to an open-ended question that they did not meet the first inclusion criterion (child returning to the same child care group as before the closure), although these parents stated that they did meet this criterion. These participants were excluded from the analyses. Participants who did not complete the questions about their own and their child’s distress during the closure and after the reopening of child care centers were excluded as well. This resulted in a final sample of 694 parents and their (youngest) child attending center-based child care. Age and gender of the target child and most child care characteristics were available for the whole sample, while family demographics were only available for 543 participants. For three variables (parental age, the number of months, and hours in child care), some impossible values were reported and therefore treated as missing, which explains the lower number of participants for these variables.

Mothers made up 90.8% of the sample and almost all parents (99.1%) were the biological parent of the child they reported about. The mean age of the children was 27.16 months (*SD* = 11.12, range = 6–52). Parents (*N* = 542) were on average 34.45 years old (*SD* = 4.28, range = 21–47). About half of the questionnaires (52%) was filled in for a boy. In 55.6% of cases, parents had more than one child. Furthermore, 96.5% of the parents and children belonged to a two-parent family. Regarding ethnicity, 93.9% of the parents were born in Netherlands and 97.1% only had the Dutch nationality. For the children, these percentages were 99.6 and 97.8%, respectively. The majority of parents (75.6%) completed their education at (applied) university level. In total, 43 parents (7.9%) indicated that their child had general health issues, such as allergies or a premature birth. Finally, 3.7% of the children had a suspected infection with COVID-19 before the reopening of child care centers and this was the case for 7% of the parents.

The mean number of months the children (*N* = 675) had attended the child care center before closure was 15.34 months (*SD* = 10.57, range = 0–43). Before closure, children were cared for at the child care center for on average 18.44 h per week (*N* = 687) and after the reopening, the number of hours per week was the same for 82.6% of the sample. The mean amount of hours at child care after reopening was slightly lower compared to before closure (*M* = 17.90, *SD* = 7.96, range = 3–44, *N* = 678). During the closure of child care centers, 33.4% of parents made use of other types of child care: in most cases children were cared for by other family members. In total, 87.9% of the children were cared for by the same professional caregivers after the reopening, while 12.1% of the children were (partially) cared for by other professional caregivers when they returned.

Power-analyses with G^∗^Power (version 3.1.9.4) showed that the sample size had to consist of a minimum of 171 participants when including 20 predictors, to find an effect size of *f*^2^ = 0.15, with a power of 0.90 and α of 0.05. Our sample size exceeded this recommended number of participants. Furthermore, the minimum number of participants for a representative sample was met as well. In total, 328,000 parents received child care allowance in 2018 in Netherlands (Rijksoverheid, 2019). The minimum number of participants for a potentially representative sample, with a confidence level of 95% and a confidence interval of 5, would therefore be 384 (which was calculated with an online tool).

### Measures

#### Child Care in Times of COVID-19: Principal Component Analysis

In consultation with a focus group of child care professionals, we constructed a questionnaire about the experiences of children and parents during the closure and after the reopening of child care centers (which we named the Child Care in Times of COVID-19 questionnaire, or in short the CiToC questionnaire, see the Supplementary Appendix for the English translation). A non-linear principal component analysis (PCA) was performed on the CiToC questionnaire to explore the potential dimensionality of this new instrument. The non-linear version of PCA was chosen because of the ordinal answering scale of the questionnaire. The PCA with Varimax rotation distinguished four different components (see below), but on theoretical grounds we decided to split one component (parental distress after reopening) into two separate components. The five final components (the two outcome variables and the first three predictors) are described below. Items with component loadings below 0.35 were not included (see the Supplementary Appendix for the subscales and items included in the current study). All subscales of the CiToC questionnaire consisted of questions that could be answered by the parent on a five-point scale with the following meanings: (1) totally disagree, (2) disagree, (3) somewhat agree, (4) agree, and (5) totally agree. Therefore, children and parents scoring 2.5 or higher on the subscales were considered to have experienced at least some distress (according to the parent). Since the questions of the CiToC questionnaire focused on distress during the first 2 weeks after the reopening, and children might have needed more time to completely readjust, we also asked parents who stated that their child displayed different behavior around drop-off and collection after the reopening compared to before the closure, how many child care days the child needed to show the same behavior as before. In an earlier report (in Dutch), which was part of the current project, we described the specific measures that were taken after the reopening of child care centers (e.g., 1.5 m distance between adults, quicker drop-off and collection), how these were received by parents and children and what behaviors (negative or positive) children displayed after the reopening (Vrijhof et al., 2020).

#### Outcomes

##### Child distress after reopening (CiToC)

Distress of the child during the first 2 weeks after the reopening of child care centers as perceived by the parent was originally assessed with 19 items. However, the PCA showed six items to load insufficiently onto the component. The other item loadings ranged from 0.58 to 0.88. Of the final 13 items, 7 items addressed the child’s reluctant behaviors toward the professional caregivers during the first day after reopening, for example: “My child did not like being touched or picked up by the professional caregivers.” The other six items focused on more general behaviors and emotions of the child during the drop-off and collection of the child at the child care center during the first 2 weeks after the reopening. An example of one of these items is: “My child was anxious when dropped off at the child care center.” Internal consistency was high (α = 0.93). An overall mean score was computed for the final selection of items. After the recoding of six items, higher mean scores indicated more child distress, as reported by the parent.

##### Parental distress after reopening (CiToC)

The subscale measuring the self-reported distress of the parent during the first 2 weeks after the reopening of child care centers consisted of seven items. All loadings were sufficient and ranged from 0.38 to 0.76. An illustration of an item is: “I found it difficult to bring my child to the child care center again.” Cronbach’s alpha was high (α = 0.86). Again, an overall mean score was computed. After the recoding of three items, higher mean scores indicated more self-reported parental distress.

#### Predictors

##### Child stress during closure (CiToC)

This subscale originally consisted of seven items, but two items loaded insufficiently, resulting in five final items. Item loadings ranged from 0.40 to 0.79. An example of an item is: “My child missed the contact with the other children at the child care center.” While answering the questions belonging to this subscale, parents were asked to think back to the closure of child care centers which ranged from the 16th of March to the 11th of May in Netherlands. When items were not applicable to their situation, parents could choose the option “not applicable.” Internal consistency was good (α = 0.80). Mean scores were only calculated if more than half of the items were valid, with higher mean scores indicating higher child stress during closure according to the parent.

##### Parental stress during closure (CiToC)

To measure parental stress during the closure of child care centers, we constructed eight items. The items again applied to the period of the first national lockdown. All but two items loaded sufficiently onto the component (range = 0.52–0.79) and were used to construct the subscale. An example of an item is: “I found it stressful to combine my caring responsibilities with my work during the closure.” When items were not applicable, parents could indicate this. Cronbach’s alpha was adequate (α = 0.76). Mean scores were only calculated if more than half of the items were valid, with higher scores indicating that the parent experienced more stress during the closure (two items were recoded for interpretation).

##### Parental fear of COVID-19 (CiToC)

According to the PCA, this subscale was part of the component “Parental stress after reopening.” However, as described, we thought it was important to distinguish the three specific items about fear of COVID-19 from the more general items about parental stress after reopening. An illustration of an item belonging to this subscale is: “I was afraid that my child would contract the coronavirus and become sick.” Loadings were 0.82, 0.80, and 0.70, and Cronbach’s alpha showed good internal consistency (α = 0.88). An overall mean score was computed for the items and higher mean scores indicated more parental fear of COVID-19.

##### Child temperament

Child temperament was measured with the validated Dutch versions of the very short form of the Infant Behavior Questionnaire-Revised (IBQ-R; Klein Velderman et al., 2006; Putnam et al., 2014) for infants under the age of 12 months, the Early Childhood Behavior Questionnaire [ECBQ; Putnam et al., 2006, translated by De Kruif, Willekens, and De Schuymer (Rothbart, 2013)] for toddlers between 12 and 36 months of age and the Children’s Behavior Questionnaire (CBQ; Putnam and Rothbart, 2006; Majdandžić and Van den Boom, 2007) for pre-schoolers older than 36 months. In these very short versions (with 36 or 37 items in total), parents are asked to indicate on a seven-point scale how often their child displayed certain behaviors during the last 7 days (IBQ-R), 14 days (ECBQ) or 6 months (CBQ). When the described situation did not occur during this period, parents could choose the “not applicable” option. Items load onto three different factors, namely “Negative Emotionality” (IBQ-R) or “Negative Affectivity” (ECBQ and CBQ), “Positive Affectivity/Surgency” (IBQ-R) or “Surgency/Extraversion” (ECBQ and CBQ), and “Orienting/Regulatory Capacity” (IBQ-R) or “Effortful Control” (ECBQ and CBQ). In the current study, we only included the subscales “Negative Emotionality/Affectivity” [α = 0.86 for IBQ-R (*N* = 36); α = 0.69 for ECBQ (*N* = 354), and α = 0.70 for CBQ (*N* = 161)] and “Positive Affectivity/Surgency/Extraversion” [α = 0.50 for IBQ-R (*N* = 36); α = 0.71 for ECBQ (*N* = 349), and α = 0.65 for CBQ (*N* = 161)]. Per subscale, a mean score was calculated, with higher scores indicating more negative affect or more extraversion. Mean scores per subscale were only calculated if more than half of the items were valid.

##### Parental separation anxiety

We used the “Maternal Separation Anxiety” subscale (MSA; 21 items) of the Maternal Separation Anxiety Scale (Hock et al., 1989) to measure the level of general parental separation anxiety. The items were adapted to fit both mothers and fathers. Furthermore, by changing phrases like “when I am away from my child” into “when my child is at the child care center,” and “than a babysitter or teacher” into “than professional caregivers,” items only relate to situations in which the child is at the child care center. We translated the items into Dutch and had them back-translated for verification by a native speaker in English who is also fluent in Dutch. Inconsistencies were discussed until consensus was reached. The questions could be answered on a five-point scale ranging from (1) strongly disagree to (5) strongly agree. The reliability analysis for the 21 items in the current study showed good internal consistency (α = 0.87, *N* = 640). Mean scores were calculated only if 75% or more of the items were answered. Higher mean scores indicated higher parental separation anxiety.

##### Parental perception of child care quality

The quality of child care from the parent’s perspective was measured with the Emlen Scales (Emlen et al., 2000). This instrument can be used in any type of child care arrangement and for children of all ages. We selected the following subscales of the larger scale “Measuring Aspects of Child Care Quality”: “Caregiver’s Warmth and Interest in my Child” (six items), “Caregiver’s Skill” (three items), and “Supportive Parent-Caregiver Relationship” (six items). The items were translated into Dutch and we had them back-translated for verification by a native speaker in English who is also fluent in Dutch. Inconsistencies were discussed until consensus was reached. We slightly changed one item from “I’m free to drop in” into “I’m free to contact,” since the latter is more common, especially in times of COVID-19. The statements could be answered on a five-point scale ranging from (1) never to (5) always. All three subscales significantly correlated with each other (range = 0.68–0.70). However, for theoretical reasons, we analyzed the subscale “Supportive Parent-Caregiver Relationship” (α = 0.84, *N* = 619) separately from the other two subscales which were combined (α = 0.90, *N* = 619), because the latter two subscales assess the interactions of professional caregivers with the child and the first the interactions of professional caregivers with the parent. Mean scores per subscale were calculated only if 75% or more of the items were answered. Higher mean scores indicate that the parent rated the child care quality more positively.

##### Other predictors

Child age (in months) at the time of the completion of the questionnaire and child hours at child care per week after the reopening of the child care centers were included as predictors as well.

##### Covariates

Potential covariates were the use of other types of child care during the closure of child care centers (yes or no), whether the child was cared for by the same professional caregivers after the reopening compared to the period before closure (yes or partly/no), the number of months the child attended the child care center before closure, the gender of the child and the parent, parental age (in years), parental educational level (low and middle levels of education vs. high level of education), family composition (one- or two-parent family), whether the parent had more than one child (yes or no), and whether the child had general health issues (yes or no).

### Multiple Imputation

As described, 151 parents had incomplete data on part of the predictor variables. In total, 9.88% of all values were missing. To check whether data imputation was recommendable, the Little’s missing completely at random (MCAR) test (Little and Rubin, 1987) was performed and proved to be non-significant [χ^2^(50324) = 40750.13, *p* = 1.000]. This meant data were missing completely at random or missing at random. We also compared the complete and non-complete groups on all complete variables. Parents who filled in the entire questionnaire scored lower on parental distress after the reopening [*M* = 2.17 vs. *M* = 2.35, *t*(692) = 2.66, *p* = 0.008], parental fear of COVID-19 [*M* = 2.04 vs. *M* = 2.27, *t*(692) = 2.85, *p* = 0.004], and higher on the number of months their child attended the child care center before closure [*M* = 15.86 vs. *M* = 13.49, *t*(673) = −2.42, *p* = 0.016] compared to parents who did not complete the entire questionnaire. Therefore, the missing values showed a pattern and were likely to be missing at random and not completely at random. Because of this finding, we chose to perform 50 multiple imputations by predictive mean matching (Markov Chain Monte Carlo) with a maximum of 50 iterations for the incomplete variables (Little and Rubin, 1987), and included all variables (covariates, predictors, and outcome variables) in the model. Missing values for the questionnaires were imputed on subscale level.

### Statistical Analysis

Two hierarchical multiple linear regression analyses on the imputed data were performed using IBM SPSS 25.0 (IBM Corp, 2017). In step one, covariates were entered. Covariates were included only if they were significantly correlated (*p* < 0.05) with the outcome variable, as evaluated in the preliminary analyses. In step two, the main predictors were entered and in step three, the concurrent stress of the parent or child (dependent on the analysis) was added. An alpha of 0.05 was used for all analyses. Pooled *F*-tests for the imputed datasets were calculated by using a macro developed by Van Ginkel (2019). Standardized regression coefficients (β’s) were averaged over the 50 imputed datasets and effect sizes (*R*^2^’s) were calculated by multiplying the mean standardized regression coefficients with the mean bivariate correlations with the outcome variable. The *R*^2^’s were subsequently summed to derive the explained variance of the models (Van Ginkel, 2020). Finally, for the purpose of a sensitivity analysis, the results of the regression analyses on the 50 imputed datasets were compared to the results for the complete cases only.

## Results

### Data Inspection

Before the main analyses were performed, we inspected the data. For five predictor variables (hours in child care, parental separation anxiety, both subscales of parental perception of child care quality, and negative affectivity) and one outcome variable (parental distress after reopening) outliers [values with a *z*-score above or below (−)3.29] were observed. Before imputation, outlying values were winsorized, such that all *z*-scores fell between −3.29 and 3.29, while retaining the original order of the data (Tabachnick and Fidell, 2013). After imputation, two to three influential cases per imputed dataset were still observed for the variables with winsorized outliers, but these cases had adequate Cook’s and leverage distances and therefore no significant impact on the regression coefficients. The residuals of both models were normally distributed and heteroscedasticity and multicollinearity were absent.

### Descriptive Statistics

In total, 52% of the parents indicated a (mostly negative) change in their child’s behavior after the reopening of child care centers compared to before the closure. According to these parents the average number of days that the children needed to readjust was 7.66 child care days (*SD* = 8.71, range = 1–60). Furthermore, 22 parents reported that their child was still not readjusted at the time when they filled out the questionnaire, which was 12.5–17 weeks after the official reopening of the child care centers. The mean level of child and parental distress after the reopening of child care centers was relatively low (*M* = 2.18 for child distress; *M* = 2.21 for parental distress). However, 29.1% of the children experienced at least some distress after the reopening (they scored on average higher than 2.5 on the scale) according to their parent, and this was the case for 31.6% of the parents. Further, 25.6% of the parents were at least somewhat afraid of COVID-19. With regard to the level of stress during the closure of child care centers we found that 73.7% of the children and 71.2% of the parents scored above the threshold. The other descriptive statistics for the complete cases are shown in Table 1.

**TABLE 1:** Descriptive statistics for the predictors and outcome variables.

	*N*	*M*	*SD*	*Min.*	*Max.*
**Outcome variables**					
Child distress after reopening^b^	694	2.18	0.83	1	4.92
Parental distress after reopening^bc^	694	2.21	0.72	1	4.71
**Predictors**					
Child age (in months)	694	27.16	11.12	6	52
Child temperament^a^					
Negative affectivity^c^	551	2.09	0.89	1	5
Surgency/extraversion	510	4.95	0.74	2.83	6.83
Child hours in child care (per week)^c^	678	17.90	7.96	3	44
Child stress during closure^b^	669	3.06	0.82	1	5
Parental stress during closure^b^	683	2.95	0.85	1	5
Parental fear of the coronavirus^b^	694	2.09	0.89	1	5
Parental separation anxiety^bc^	640	2.24	0.44	1	3.75
Parental perception of child care quality^b^					
Caregiver’s warmth and interest in the child and caregiver’s skill (child)^c^	619	4.41	0.46	2.34	5
Supportive parent-caregiver relationship (parent)^c^	619	4.24	0.59	2.16	5

*Descriptive statistics for complete cases.*

*^a^Answering scale ranged from 1 to 7 [composite mean score of infant behavior questionnaire-revised (IBQ-R), early childhood behavior questionnaire (ECBQ), and children’s behavior questionnaire (CBQ)].*

*^b^Answering scale ranged from 1 to 5.*

*^c^Winsorized.*

### Bivariate Correlational Analyses

In Table 2, the bivariate correlations among the predictors, outcome variables and covariates for both the pooled and complete cases are displayed. Because four dichotomous covariates (child health, family type, parental gender, and caregiver stability) had unequal distributions over the categories, we compared the outcomes of the regular correlations with the outcome variables with correlations based on 1,000 bootstrap samples. No differences were found, showing the correlations to be robust. As one can see in Table 2, higher levels of child and parental distress were related to a lower number of months in child care before closure, lower stability of the professional caregivers and single-parent families. Furthermore, a higher level of child distress was related to a higher parental educational level and mothers scored higher on parental distress than fathers. These variables were therefore included in the analysis as covariates.

**TABLE 2:** Bivariate correlations among the predictors, outcome variables, and covariates.

	1	2	3	4	5	6	7	8	9	10	11	12	13	14	15	16	17	18	19	20	21	22
1. Child age	–	0.302**	−351**	−0.054	0.126**	−0.141**	−0.076	−0.101*	0.017	−0.026	−0.246**	−0.084*	0.005	0.036	0.768**	−0.039	−0.042	−0.03	0.198**	−0.102*	−0.066	0.236**
2. Negative affectivity	0.295**	–	−0.385**	0.006	0.117**	0.196**	−0.149**	−0.101*	0.088*	0.112**	0.068	0.095*	0.099*	0.045	0.159**	−0.052	−0.003	−0.02	0.098*	−0.104*	0.006	0.109*
3. Surgency/extraversion^a^	−0.321**	−0.359**	–	0.005	0.162**	−0.061	0.195**	0.166**	0.037	−0.068	−0.061	−0.117**	−0.078	0.021	−0.230**	0.135**	0.057	0.014	−0.154**	−0.038	−0.002	−0.205**
4. Child hours in child care	−0.058	0.025	−0.013	–	0.135**	−0.127**	−0.014	−0.006	0.289**	−0.099**	−0.101**	−0.129**	−0.024	−0.039	0.108**	−0.024	−0.039	−0.180**	−0.047	0.144**	−0.081	0.173**
5. Child stress during closure	0.129**	0.111**	0.148**	0.135**	–	−0.115**	0.119**	0.089*	0.326**	0.009	−0.223**	−0.209**	0.044	0.083	0.112**	0.063	0.045	−0.044	−0.114**	−0.168**	0.058	−0.127**
6. Parental separation anxiety	−0.135**	. 199**	−0.058	−0.116**	−0.112**	–	−0.359**	−0.237**	−0.076	0.297**	0.468**	0.553**	0.043	0.084*	−0.161**	−0.079*	−0.089*	−0.08	−0.051	−0.064	0.109*	−0.094*
7. Parental perception of child care quality—child	−0.073	−0.151**	0.189**	−0.016	0.116**	−0.351**	–	0.743**	−0.043	−0.082*	−0.270**	−0.217**	−0.009	−0.013	−0.055	0.099*	0.087*	0.015	−0.026	−0.01	0.049	−0.126**
8. Parental perception of child care quality—parent	−0.101*	−0.099*	0.157**	−0.005	0.088*	−0.232**	0.740**	–	−0.099*	−0.071	−0.206**	−0.101*	−0.028	0.012	−0.095*	0.078	0.106*	0.007	−0.077	−0.054	0.056	−0.135**
9. Parental stress during closure	0.013	0.082	0.02	0.292**	0.331**	−0.064	−0.041	−0.095*	–	−0.045	0.027	−0.234**	−0.058	0.103*	0.114**	−0.104**	−0.019	−0.03	0.063	0.105*	0.035	0.186**
10. Parental fear of coronavirus	−0.026	0.102*	−0.052	−0.105**	0.008	0.297**	−0.079*	−0.07	−0.043	–	0.071	0.458**	−0.04	0.054	−0.066	−0.07	−0.118**	−0.056	−0.008	−0.021	0.098*	−0.02
11. Child distress after reopening	−0.246**	0.073	−0.07	−0.098*	−0.221**	0.456**	−0.261**	−0.203**	0.027	0.071	–	0.347**	−0.058	0.034	−0.182**	−0.01	−0.115**	−0.107*	−0.016	0.095*	0.090*	−0.023
12. Parental distress after reopening	−0.084*	0.085*	−0.107*	−0.132**	−0.206**	0.547**	−0.209**	−0.094*	−0.235**	0.458**	0.347**	–	−0.015	0.076	−0.096*	−0.065	−0.129**	−0.089*	0.003	0.031	0.153**	−0.043
13. Child gender^b^	0.005	0.087*	−0.067	−0.019	0.045	0.042	−0.015	−0.032	−0.056	−0.04	−0.058	−0.015	–	−0.036	0.008	0.035	0.029	−0.003	−0.009	−0.031	−0.012	−0.001
14. Child general health^c^	0.031	0.038	0.012	−0.037	0.072	0.078	−0.013	0.01	0.094*	0.058	0.032	0.076	−0.038	–	−0.003	0.036	−0.064	−0.018	0.001	−0.056	0.023	−0.120*
15. Number of months in child care before closure	0.766**	0.146**	−0.213**	0.100**	0.114**	−0.164**	−0.053	−0.094*	0.110**	−0.062	−0.186**	−0.099*	0.009	−0.005	–	−0.073	−0.061	0.01	0.167**	0.033	−0.055	0.248**
16. Use of other forms of child care during closure	−0.039	−0.048	0.139**	−0.02	0.062	−0.086*	0.095*	0.072	−0.107**	−0.07	−0.01	−0.065	0.035	0.033	−0.068	–	−0.018	−0.034	−0.116**	−0.214**	0.119**	−0.204**
17. Stability of professional caregivers^d^	−0.042	−0.005	−0.05	−0.039	0.043	−0.083*	0.080*	0.101*	−0.026	−0.118**	−0.115**	−0.129**	0.029	−0.07	−0.06	−0.018	–	0.087*	0.047	−0.044	0.004	−0.019
18. Family composition^e^	−0.037	−0.02	0.025	−0.198**	−0.027	−0.093*	0.026	0.011	−0.024	−0.055	−0.094*	−0.086*	−0.004	−0.018	0.001	−0.024	0.095*	–	0.153**	0.102*	−0.061	0.043
19. Number of children^f^	0.191**	0.088*	−0.144**	−0.049	−0.106*	−0.06	−0.012	−0.064	0.057	−0.013	−0.017	0.003	−0.017	0.001	0.147**	−0.114**	0.059	0.144**	–	0.064	−0.067	0.307**
20. Parental education^g^	−0.096*	−0.104*	−0.059	0.140**	−0.149**	−0.064	−0.006	−0.043	0.104*	−0.012	0.084*	0.035	−0.032	−0.049	0.037	−0.204**	−0.043	0.098*	0.059	–	−0.032	0.189**
21. Parental gender^b^	−0.06	0.003	−0.002	−0.08	0.057	0.090*	0.056	0.061	0.035	0.094*	0.07	0.141**	−0.017	0.018	−0.05	0.108**	0.004	−0.048	−0.063	−0.023	–	−0.209**
22. Parental age	0.229**	0.101*	−0.192**	0.174**	−0.124**	−0.087*	−0.112**	−0.117**	0.178**	−0.022	−0.011	−0.041	−0.004	−0.105*	0.233**	−0.200**	−0.016	0.029	0.297**	0.177**	−0.200**	–

*Correlations under the diagonal were pooled for all 50 imputed datasets (*N* = 694). Correlations above the diagonal were calculated for complete cases only.*

*p < 0.05, **p < 0.01 (two-tailed).

*^a^Composite mean score for infant behavior questionnaire-revised (IBQ-R), early childhood behavior questionnaire (ECBQ), and Children’s Behavior Questionnaire (CBQ).*

*^b^0 = boy/male, 1 = girl/female.*

*^c^0 = good, 1 = health issues.*

*^d^0 = no, 1 = yes.*

*^e^1 = one-parent family, 2 = two-parent family.*

*^f^1 = one child, 2 = more than one child.*

*^g^1 = low/middle, 2 = high.*

### Hierarchical Multiple Linear Regression Analysis: Child Distress After Reopening

The regression analysis for child distress was performed in three steps. All three models were significant (*p* < 0.001) and the third model was significantly better than the first and second model (*p* < 0.001). The final model (Table 3; Model 3) had an explained variance of 34.4% and showed that younger children (β = −0.29, *p* < 0.001) and children with parents scoring higher on separation anxiety (β = 0.29, *p* < 0.001) experienced more distress after the reopening. Furthermore, children with parents who reported more distress after reopening (β = 0.17, *p* < 0.001), children who spent less hours at the child care center after reopening (β = −0.13, *p* < 0.001), children who experienced less stress during closure according to their parent (β = −0.13, *p* < 0.001), and children from one-parent families (β = −0.09, *p* = 0.012) were more distressed upon return. The regression coefficients for parental fear of COVID-19 (β = −0.13, *p* < 0.001) and parental stress during closure (β = 0.14, *p* < 0.001) also reached significance, but these coefficients were not in line with the non-significant bivariate correlations (*r* = 0.07 and *r* = 0.03, respectively), indicating negative suppression (Tabachnick and Fidell, 2013). Therefore, these predictors seemed to add to the model, but could not be considered sound predictors in itself.

**TABLE 3:** Results of the hierarchical multiple linear regression analysis predicting child distress after reopening (*N* = 694).

	Step 1				Step 2				Step 3			
	*B*	*SE*	β	*t*	*B*	*SE*	β	*t*	*B*	*SE*	β	*t*
(Intercept)	3.15	0.39		8.00**	3.51	0.61		5.79**	3.22	0.61		5.29**
Number of months in child care before closure	−0.02	0.00	−0.20	−5.30**	0.01	0.00	0.08	1.45	0.01	0.00	0.07	1.32
Stability of professional caregivers^a^	−0.29	0.10	−0.11	−3.07**	−0.19	0.08	−0.07	−2.24*	−0.16	0.08	−0.06	–1.99
Family composition^b^	−0.41	0.19	−0.09	−2.13*	−0.44	0.16	−0.10	−2.69**	−0.41	0.16	−0.09	−2.50*
Parental educational level^c^	0.19	0.08	0.10	2.36*	0.13	0.07	0.07	1.90	0.11	0.07	0.06	1.59
Child age					−0.02	0.00	−0.30	−5.16**	−0.02	0.00	−0.29	−5.10**
Negative affectivity					0.06	0.04	0.06	1.53	0.07	0.04	0.07	1.62
Surgency/extraversion					−0.07	0.05	−0.06	–1.39	−0.06	0.05	−0.05	–1.20
Child hours in child care					−0.01	0.00	−0.14	−3.79**	−0.01	0.00	−0.13	−3.74**
Child stress during closure					−0.14	0.04	−0.14	−3.82**	−0.13	0.04	−0.13	−3.49**
Parental separation anxiety					0.69	0.07	0.36	9.48**	0.56	0.08	0.29	6.91**
Parental perception of child care quality—child					−0.15	0.10	−0.08	–1.54	−0.12	0.10	−0.06	–1.23
Parental perception of child care quality—parent					−0.05	0.07	−0.04	–0.74	−0.08	0.07	−0.06	–1.10
Parental stress during closure					0.12	0.04	0.11	2.92**	0.13	0.04	0.14	3.67**
Parental fear of coronavirus					−0.08	0.03	−0.08	−2.37**	−0.13	0.03	−0.13	−3.72**
Parental distress after reopening									0.20	0.05	0.17	3.95**
*R* ^2^	0.07**				0.33**				0.34**			
*F*_(__*df*__1__, df__2__)_	*F*_(__4, 649)_ = 10.75**				*F*_(__14, 668)_ = 21.07**				*F*_(__15, 669)_ = 21.22**			

*Regression coefficients were pooled for all 50 imputed datasets.*

*B, regression coefficient; *SE*, standard error; β, beta coefficient or standardized regression coefficient; *t*, *t*-value; *R*^2^, coefficient of determination; *F*_(__*df*__1__, df__2__)_, *F*-value and degrees of freedom.*

*p < 0.05, **p < 0.01.

*^a^0 = no, 1 = yes.*

*^b^1 = one-parent family, 2 = two-parent family.*

*^c^1 = low/middle, 2 = high.*

### Hierarchical Multiple Linear Regression Analysis: Parental Distress After Reopening

The regression analysis for parental distress was also performed in three steps, and again, all three models were significant (*p* < 0.001). The final model (Table 4; Model 3), significantly better than the first and second model (*p* < .001), explained 47.6% of the variance and showed that parents scoring higher on general separation anxiety (β = 0.36, *p* < 0.001) and fear of COVID-19 (β = 0.33, *p* < 0.001) experienced more distress after reopening. Moreover, parents experiencing less stress during closure (β = −0.18, *p* ≤ 0.001), parents of children experiencing more stress after reopening (β = 0.14, *p* ≤ 0.001) as well as less during closure (β = −0.07, *p* = 0.022), and mothers (β = 0.08, *p* = 0.017) also experienced more distress. Again, one predictor reached significance (*p* = 0.021), but did not match the negative bivariate correlation, which could be attributed to a negative suppressor effect (Tabachnick and Fidell, 2013). This predictor was the parental perception of child care quality toward the parent (β = 0.10 vs. *r* = −0.09).

**TABLE 4:** Results of the hierarchical multiple linear regression analysis predicting parental distress after reopening (*N* = 694).

	Step 1				Step 2				Step 3			
	*B*	*SE*	β	*t*	*B*	*SE*	β	*t*	*B*	*SE*	β	*t*
(Intercept)	2.76	0.34		8.18**	1.56	0.49		3.208**	1.12	0.50		2.238*
Number of months in child care before closure	−0.01	0.00	−0.10	−2.64**	0.00	0.00	0.06	1.19	0.00	0.00	0.05	0.95
Stability of professional caregivers^a^	−0.29	0.08	−0.13	−3.468**	−0.12	0.06	−0.05	–1.87	−0.10	0.06	−0.04	–1.53
Family composition^b^	−0.26	0.16	−0.07	–1.59	−0.11	0.14	−0.03	–0.79	−0.06	0.14	−0.02	–0.44
Parental gender^c^	0.34	0.10	0.13	3.22**	0.213	0.09	0.08	2.51*	0.20	0.09	0.08	2.39*
Child age					−0.00	0.00	−0.06	–1.11	−0.00	0.00	−0.02	–0.29
Negative affectivity					−0.02	0.03	−0.02	–0.56	−0.03	0.03	−0.03	–0.78
Surgency/extraversion					−0.05	0.04	−0.06	–1.38	−0.05	0.04	−0.05	–1.17
Child hours in child care					0.00	0.00	0.00	–0.04	0.00	0.00	0.02	0.49
Child stress during closure					−0.09	0.03	−0.10	−2.96**	−0.07	0.03	−0.07	−2.29*
Parental separation anxiety					0.67	0.06	0.41	−11.72**	0.58	0.06	0.36	9.78**
Parental perception of child care quality—child					−0.16	0.07	−0.10	−2.20*	−0.14	0.07	−0.09	–1.98
Parental perception of child care quality—parent					0.12	0.06	0.10	2.15*	0.13	0.05	0.10	2.31*
Parental stress during closure					−0.14	0.03	−0.17	−5.15**	−0.15	0.03	−0.18	−5.67**
Parental fear of coronavirus					0.25	0.02	0.31	10.36**	0.26	0.02	0.33	10.81**
Child distress after reopening									0.12	0.03	0.14	4.04**
*R* ^2^	0.05**				0.46**				0.48**			
*F*_(__*df1, df2)*_	*F*_(__4, 652)_ = 8.18**				*F*_(__14, 661)_ = 35.45**				*F*_(__15, 662)_ = 35.13**			

*Regression coefficients were pooled for all 50 imputed datasets.*

*B, regression coefficient; *SE*, standard error; β, beta coefficient or standardized regression coefficient; *t*, *t*-value; *R*^2^, coefficient of determination; *F*_(__*df*__1__, df__2__)_, *F*-value and degrees of freedom.*

*p < 0.05, **p < 0.01.

*^a^0 = no, 1 = yes.*

*^b^1 = one-parent family, 2 = two-parent family.*

*^c^0 = male, 1 = female.*

### Sensitivity Analysis

Compared to the pooled results, the outcomes of the analyses with complete cases only (*N* = 543) showed some differences (see Supplementary Tables S1, S2). The final model for child distress after reopening indicated that negative affectivity was a significant predictor (β = 0.12, *p* = 0.009), while this was not the case for the analysis that included cases with imputed data. For parental distress after reopening two differences were found, the first of which concerned parental perception of child care quality toward the child, which significantly contributed to the model for the complete cases (β = −0.13, *p* = 0.015), but not for the analysis making use of imputations. Furthermore, the level of child stress during closure (β = −0.06, *p* = 0.116) was not a significant predictor in the model for the complete cases, while it was for the model including the imputed data. These differences indicate that multiple imputation was justified, as the outcomes were slightly different for some of the predictors.

## Discussion

In the current paper we studied what factors contributed to variance in child and parental distress during the reopening of child care centers after a 2-month lockdown because COVID-19. Results indicated that about one-third of the children (29.1%) and parents (31.6%) experienced distress upon the child’s return to the child care center, as reported by the parent. An explanation for the apparent discrepancy between the percentage for child distress after reopening and the percentage regarding the children that displayed different behavior after the reopening (52%), is that for the subscale on child distress, parents needed to report on average at least some distress (2.5 or higher), while the single question about behavior also applied to minor changes regarding one specific behavior (e.g., crying). Moreover, 25.6% of the parents reported that they were (somewhat) afraid of COVID-19 around the reopening. During closure, 73.7% of the children and 71.2% of the parents experienced at least some stress, as perceived by the parent. Thus, parents and children were more distressed during the closure than after the reopening of child care centers, at least according to parental (self-)report. The disadvantages of closed facilities might have weighed heavier than the difficulties around the reopening for most children and parents. The strongest predictors of child distress upon return were child age and parental separation anxiety, with younger children and children with parents experiencing more separation anxiety showing more distress after the reopening. Furthermore, concurrent parental distress was positively associated with child distress, and child hours spent in child care and child distress during closure were significant negative predictors. Finally, children from one-parent families experienced more distress upon return than children from two-parent families. In parents, parental separation anxiety and parental fear of COVID-19 explained most of the variance in their distress, with parents scoring higher on separation anxiety and fear of COVID-19 experiencing more distress when their child re-entered the child care center. Moreover, we found that mothers experienced more distress, as well as parents with lower stress levels during the closure and parents with more distressed children upon return.

### Explaining Differences in Child Distress After Reopening

Child distress after reopening was significantly associated with several child, parental and COVID-19 related factors. First, the results of the current study showed younger children to experience more distress when returning to the child care center than older children, as reported by the parent. Younger children have less self-regulatory capacities and were found to be more susceptible to stressors (Gunnar and Donzella, 2002), and therefore might experience more distress around transitions. It should be noted though that most children in the younger age range in the current sample were around 12 months of age and none of the children were younger than 6 months. Therefore, the negative relation between child age and distress upon return might be partly explained by the occurrence of separation anxiety in children as part of a healthy development between 6 and 18 months, when children’s attachment bonds with their primary caregivers are being consolidated (Schaffer and Emerson, 1964). Other important predictors of child distress upon return were parental separation anxiety and parental distress after reopening. This positive link between parental emotional reactions and child functioning has been described as a cross-over effect, in which the emotional reactions of one person within a subsystem influence the emotional reactions of another person (Nelson et al., 2009). The underlying mechanism that has been proposed for explaining this relation is parenting behavior (Deater-Deckard, 1998), although some studies only found a direct effect of parenting stress on child functioning (e.g., Crnic et al., 2005). In the current study, anxious or overprotective parenting could be an explanatory mechanism, although this is a speculation. The hours spent in child care per week were negatively associated with child distress after reopening. Spending more hours at the child care center after an interruption may be beneficial for the adjustment process, because this may help children to get used more easily to the child care setting. It should be noted that in the current study, most children (around 80%) spent 1–4 days at the child care center, which is common in the Netherlands, where full-time child care is an exception. Another predictor was child stress during the closure of child centers: children who missed the child care center to a larger extent during closure, showed less distress upon return. It appears that children who missed the child care center were more excited to return after the reopening and therefore might have experienced less distress, according to their parent. Finally, children from one-parent families experienced more distress when they returned to the child care center. Although family composition was not a predictor of parental distress, children in one-parent families might experience more distress upon return because in general, single parents experience more parenting stress than parents with a partner (Copeland and Harbaugh, 2005), and this parental stress might have crossed over to the child.

Contrary to our expectations, child temperament and child care quality as perceived by the parent were no significant predictors of child distress. We, however, did find negative affectivity to be a significant predictor for the complete cases, with a small difference in the beta weight compared to the pooled data. Since parents who partially filled out the questionnaire reported more parental distress upon return, speculatively, child factors such as negative affect might be only a significant predictor when parental factors such as parental distress are less dominant. When parents experience distress above a certain threshold, the effect of child negative affectivity on child distress may vanish, as parental distress might have a larger impact. Regarding temperament, this variable may act as a moderator, as was for example found in the study by Albers et al. (2016). This study showed higher afternoon cortisol levels during the first weeks at child care for infants who received higher quality of maternal care, but only if infants also scored higher on negative emotionality.

### Explaining Differences in Parental Distress After Reopening

Parental factors contributed more to the explained variance in parental distress after reopening than child factors. This is in line with the conclusion by Cobham et al. (2016), who found child functioning to predict parental outcomes only in a minority of studies. As expected, parental separation anxiety and parental fear of COVID-19 were positively associated with parental distress. Comparable results were found in other recent studies into stress and parenting during the COVID-19 pandemic. For example, Brown et al. (2020) found COVID-19 related stressors, and high anxiety and depressive symptoms to correlate with higher parental stress. We additionally found evidence for a negative relation between parental stress during closure and parental distress after reopening, which might be explained by the relieve that parents who experienced more stress during closure might have felt when they were able to bring their child to the child care center after 2 months. In the current study, mothers experienced more distress than fathers, which corresponds with the general finding that women suffer more from anxiety and depressive symptoms than men (Faravelli et al., 2013), and which was also found in a recent study on lockdown-related traumatic stress in parents (Chartier et al., 2021). We also found that child distress was a significant predictor of parental distress. More research is needed to further explore the (bi)directionality of the relation between child and parental distress. Finally, results showed that child care quality as perceived by the parent did not predict parental distress, which contradicted our expectation. The absence of this effect might be explained by the limited variation in child care quality overall, as parents rated the child care quality of the child care setting rather positive.

### Limitations and Implications

The current study showed that about one-third of the parents and children experienced distress when the child returned to the child care center after the lockdown (as perceived by the parent). When discussing these results it is, however, important to note that after the reopening, child care centers took several measures to minimize the spread of COVID-19, such as keeping 1.5 m distance between all adults and a quicker drop-off and collection at the door. As we described in a previous report (Vrijhof et al., 2020), these measures were mainly perceived as negative by parents, both for their children and themselves, and therefore it is likely that these measures have had an impact on experienced parental and child distress after reopening. The effect of the interruption of care and the effect of the measures after reopening are difficult to disentangle, but unfortunately inherent to the situation. Further, as our preliminary analyses showed, parents who filled out the questionnaire partially, scored higher on distress upon return to the child care center than parents who completed the entire questionnaire. Therefore, it could be that the most stressed parents did not fill out the questionnaire at all, leading to an underestimation of the levels of distress upon return. However, the relatively low levels of distress could also reflect reality, as other studies also found that only a minority responded negatively to the reopening of schools and work places after a lockdown during the COVID-19 pandemic (Gilbert et al., 2020; Tan et al., 2020). Another limitation concerns the possibility that some questions in the current study could have elicited socially desirable answers, especially with regard to the CiToC questionnaire. Parents might have not wanted to report extremely low or high distress, as the first may be seen as indifference and the second as conflicting with bringing their child to the child care center.

In addition, the retrospective and one-informant design of the study has some drawbacks. First, parents reported about both their own and their child’s distress, which could have resulted in parents with distress over-reporting their child’s distress, as was found in other studies (Briggs-Gowan et al., 1996). However, the relatively low bivariate correlation between and different results for the two outcome variables give confidence in the independent rating of constructs. Secondly, parents filled out the questionnaire several months after the actual reopening of child care centers and their memories may have faded somewhat or decreased in intensity. However, because of the extraordinary nature of events, it was not possible to distribute the questionnaire earlier and we expected parents to remember the details rather accurately because of this. Moreover, it was mentioned that participants could discuss the CiToC questionnaire with their partner to increase validity. Finally, all variables were measured simultaneously, and conclusions regarding causality can therefore not be drawn. Future studies should ideally implement a multi-informant (including both parents and professional caregivers), multi-method design (including both questionnaires and observations) and follow children and parents prospectively over time as they (re)transition into a (new) child care setting, and further explore the proposed underlying mechanisms of the association. Additionally, such a design could help to answer questions about how long children and parents need to (re)adjust and what factors account for variation in the length of this process. Related factors such as family socio-economic status and social support should be included as well, as these can (partly) influence other factors, such as the number of hours that the child spends at the child care center (which was found to be related to child distress).

As widespread closures of child care centers might happen again, not only because of the COVID-19 pandemic, the outcomes of the current paper can give direction to policy makers and professionals in comparable future situations. The interruption of care can be related to large-scale disasters, but also to individual circumstances, such as hospitalization of the child. After such an interruption, extra attention should be directed to younger children, children spending less hours at child care (children of) parents with higher levels of parental separation anxiety, and parents with higher levels of situation-specific anxieties (in this case: more fear of COVID-19). Considering the strength of the association between parental separation anxiety and both outcome measures, it would be interesting to investigate what parents and professional caregivers think about the feasibility of the MSA subscale (Hock et al., 1989) as a screening instrument. The questionnaire might then help professionals to identify children and parents that may be in need of some extra support. However, this idea raises some ethical questions that should be discussed first, for example who would get access to this personal information. Furthermore, research into useful cut-off scores would be needed then. In the meantime, child care organizations could think of encouraging professional caregivers to communicate with parents before the return and ask them about their feelings and potential worries regarding the interruption of care and the return of the child to the child care center. Another implication is that few hours in child care per week might be less beneficial for the adjustment process of children. Whether certain thresholds exist regarding the amount of hours that is necessary for a smooth (re)transition should be studied in future research. A final avenue for prospective research concerns studying the types of support that help children and parents best with making a smooth (re)transition into the child care setting.

## Conclusion

The current study demonstrated that child age, child hours in child care, child and parental stress during closure, parental separation anxiety, parental fear of COVID-19, parental gender, and family composition are predictors of child and parental distress when the child returned to the child care center after a 2-month national lockdown. Especially younger children, children spending less hours at child care and (children of) anxious parents could benefit from some extra support when they return after an interruption. Communicating with parents about potential worries regarding the return of children is crucial to be able to identify these families. Future research should use prospective designs in which the observations of multiple informants are included and the underlying mechanisms, such as parenting practices, of the observed associations are studied.

## Data Availability Statement

The datasets presented in this article are not readily available because if we receive a request to share our data the lead investigator HV, possibly in consultation with the other members of the research team, will decide whether or not to agree with this request. We support the notion that other researchers must be able to verify results and that it can be worthwhile to reuse data for future research. Requests to access the datasets should be directed to HV, vermeer@fsw.leidenuniv.nl.

## Ethics Statement

The studies involving human participants were reviewed and approved by the Ethics Committee of the Institute of Education and Child Studies of Leiden University. The participants provided their written informed consent to participate in this study.

## Author Contributions

SMV, CV, and JP coordinated the data collection. SMV and CV organized the database. SMV wrote the initial draft and conducted the statistical analyses. All authors contributed to the conception and design of the study, manuscript revision, read, and approved the submitted version.

## Conflict of Interest

The authors declare that the research was conducted in the absence of any commercial or financial relationships that could be construed as a potential conflict of interest.

## Publisher’s Note

All claims expressed in this article are solely those of the authors and do not necessarily represent those of their affiliated organizations, or those of the publisher, the editors and the reviewers. Any product that may be evaluated in this article, or claim that may be made by its manufacturer, is not guaranteed or endorsed by the publisher.
