# Supplementary material for: Child Care in Times of COVID-19: Predictors of Distress in Dutch Children and Parents When Re-entering Center-Based Child Care After a 2-Month Lockdown
Source: Front Psychol. 2021 Nov 5;12:718898. doi: 10.3389/fpsyg.2021.718898 (PMC8603750; doi:10.3389/fpsyg.2021.718898)
Supplement: Supplementary file 1 [file Table_1.docx]

**Table S1**

*Results of the Hierarchical Multiple Linear Regression Analysis Predicting Child Distress after Reopening for the Complete Cases (N = 543)*

|  | **Step 1** | | | | | | | |  | **Step 2** | | | | | | | |  | **Step 3** | | | | | | | |
| --- | --- | --- | --- | --- | --- | --- | --- | --- | --- | --- | --- | --- | --- | --- | --- | --- | --- | --- | --- | --- | --- | --- | --- | --- | --- | --- |
|  | *B* | | *SE* | | *β* | | *t* | |  | *B* | | *SE* | | *β* | | *t* | |  | *B* | | *SE* | | *β* | | *t* | |
| (Intercept) | 3.47 | | 0.45 | |  | | 7.72** | |  | 3.90 | | 0.70 | |  | | 5.55** | |  | 3.46 | | 0.70 | |  | | 4.95** | |
| Number of months in child care before closure | -0.02 | | 0.00 | | -.25 | | -5.62** | |  | 0.01 | | 0.00 | | .06 | | 1.07 | |  | 0.00 | | 0.00 | | .04 | | 0.67 | |
| Stability of professional caregivers^a^ | -0.27 | | 0.11 | | -.11 | | -2.41* | |  | -0.19 | | 0.10 | | -.08 | | -2.00 | |  | -0.16 | | 0.09 | | -.06 | | -1.71 | |
| Family composition^b^ | -0.52 | | 0.22 | | -.11 | | -2.42* | |  | -0.50 | | 0.19 | | -.10 | | -2.65** | |  | -0.46 | | 0.18 | | -.10 | | -2.49* | |
| Parental educational level^c^ | 0.20 | | 0.09 | | .10 | | 2.16* | |  | 0.16 | | 0.08 | | .08 | | 1.97 | |  | 0.13 | | 0.08 | | .07 | | 1.70 | |
| Child age |  |  | |  | |  | |  |  | -0.03 | | 0.01 | | -.39 | | -6.01** | |  | -0.03 | | 0.01 | | -.36 | | -5.70** | |
| Negative affectivity |  |  | |  | |  | |  |  | 0.13 | | 0.05 | | .12 | | 2.61** | |  | 0.13 | | 0.05 | | .12 | | 2.61** | |
| Surgency/Extraversion |  |  | |  | |  | |  |  | -0.08 | | 0.05 | | -.07 | | -1.61* | |  | -0.07 | | 0.05 | | -.06 | | -1.37 | |
| Child hours in child care |  |  | |  | |  | |  |  | -0.01 | | 0.00 | | -.10 | | -2.37** | |  | -0.01 | | 0.00 | | -.10 | | -2.37* | |
| Child stresss during closure |  |  | |  | |  | |  |  | -0.15 | | 0.04 | | -.14 | | -3.41** | |  | -0.14 | | 0.04 | | -.13 | | -3.09** | |
| Parental separation anxiety |  |  | |  | |  | |  |  | 0.69 | | 0.09 | | .36 | | 8.16 | |  | 0.51 | | 0.09 | | .27 | | 5.48** | |
| Parental perception of child care quality – Child |  |  | |  | |  | |  |  | -0.16 | | 0.11 | | -.08 | | -1.47 | |  | -0.10 | | 0.11 | | -.05 | | -0.97 | |
| Parental perception of child care quality – Parent |  |  | |  | |  | |  |  | -0.05 | | 0.08 | | -.04 | | -0.65 | |  | -0.09 | | 0.08 | | -.06 | | -1.13 | |
| Parental stress during closure |  |  | |  | |  | |  |  | 0.05 | | 0.04 | | .05 | | 1.21 | |  | 0.09 | | 0.04 | | .09 | | 2.15* | |
| Parental fear of coronavirus |  |  | |  | |  | |  |  | -0.05 | | 0.04 | | -.05 | | -1.29 | |  | -0.11 | | 0.04 | | -.11 | | -2.64** | |
| Parental distress after reopening |  |  | |  | |  | |  |  |  |  | |  | |  | |  |  | 0.26 | | 0.06 | | .21 | | 4.23** | |
| *R^2^* | .09** |  | |  | |  | |  |  | .39** |  | |  | |  | |  |  | .41** |  | |  | |  | |  |
| *F*(df_1_, df_2_) | *F*(4, 456) = 11.86)** | | | | | | | | | *F*(14, 446) = 20.03** | | | | | | | |  | *F*(15, 445) = 20.59** | | | | | | | |

*Note. B* = regression coefficient, *SE* = standard error, *β* = beta coefficient or standardized regression coefficient, *t* = t-value; *R^2^* = coefficient of determination;

*F*(df_1_, df_2_) = F-value and degrees of freedom.

* *p* < .05, ** *p* < .01, ^a^ 0 = no, 1 = yes, ^b^ 1 = one-parent family, 2 = two-parent family, ^c^ 1 = low/middle, 2 = high

**Table S2**

*Results of the Hierarchical Multiple Linear Regression Analysis Predicting Parental Distress after Reopening for the Complete Cases (N = 543)*

|  | **Step 1** | | | | | | | |  | **Step 2** | | | | | | | |  | **Step 3** | | | | | | | |
| --- | --- | --- | --- | --- | --- | --- | --- | --- | --- | --- | --- | --- | --- | --- | --- | --- | --- | --- | --- | --- | --- | --- | --- | --- | --- | --- |
|  | *B* | | *SE* | | *β* | | *t* | |  | *B* | | *SE* | | *β* | | *t* | |  | *B* | | *SE* | | *β* | | *t* | |
| (Intercept) | 2.62 | | 0.38 | |  | | 6.86** | |  | 1.76 | | 0.53 | |  | | 3.32** | |  | 1.15 | | 0.54 | |  | | 2.14* | |
| Number of months in child care before closure | 0.00 | | 0.00 | | -.07 | | -1.53 | |  | 0.01 | | 0.00 | | .12 | | 2.20* | |  | 0.01 | | 0.00 | | .10 | | 1.99 | |
| Stability of professional caregivers^a^ | -0.26 | | 0.10 | | -.13 | | -2.75** | |  | -0.12 | | 0.07 | | -.06 | | -1.65 | |  | -0.09 | | 0.07 | | -.04 | | -1.27 | |
| Family composition^b^ | -0.24 | | 0.18 | | -.06 | | -1.35 | |  | -0.10 | | 0.14 | | -.03 | | -0.71 | |  | -0.04 | | 0.14 | | -.01 | | -0.25 | |
| Parental gender^c^ | 0.38 | | 0.11 | | .16 | | 3.47** | |  | 0.21 | | 0.08 | | .09 | | 2.49* | |  | 0.18 | | 0.08 | | .08 | | 2.24* | |
| Child age |  |  | |  | |  | |  |  | -0.01 | | 0.00 | | -.12 | | -1.99 | |  | 0.00 | | 0.00 | | -.05 | | -0.78 | |
| Negative affectivity |  |  | |  | |  | |  |  | 0.00 | | 0.04 | | .00 | | 0.11 | |  | -0.01 | | 0.04 | | -.02 | | -0.37 | |
| Surgency/Extraversion |  |  | |  | |  | |  |  | -0.05 | | 0.04 | | -.05 | | -1.31 | |  | -0.04 | | 0.04 | | -.04 | | -1.00 | |
| Child hours in child care |  |  | |  | |  | |  |  | 0.00 | | 0.00 | | .01 | | 0.19 | |  | 0.00 | | 0.00 | | .02 | | 0.59 | |
| Child stress during closure |  |  | |  | |  | |  |  | -0.08 | | 0.03 | | -.09 | | -2.32* | |  | -0.05 | | 0.03 | | -.06 | | -1.58 | |
| Parental separation anxiety |  |  | |  | |  | |  |  | 0.68 | | 0.07 | | .43 | | 10.42** | |  | 0.58 | | 0.07 | | .37 | | 8.48** | |
| Parental perception of child care quality – Child |  |  | |  | |  | |  |  | -0.22 | | 0.08 | | -.14 | | -2.71** | |  | -0.20 | | 0.08 | | -.13 | | -2.45* | |
| Parental perception of child care quality – Parent |  |  | |  | |  | |  |  | 0.14 | | 0.06 | | .12 | | 2.29* | |  | 0.15 | | 0.06 | | .12 | | 2.48* | |
| Parental stress during closure |  |  | |  | |  | |  |  | -0.15 | | 0.03 | | -.19 | | -4.83** | |  | -0.16 | | 0.03 | | -.20 | | -5.18** | |
| Parental fear of coronavirus |  |  | |  | |  | |  |  | 0.22 | | 0.03 | | .27 | | 7.37** | |  | 0.23 | | 0.03 | | .28 | | 7.74** | |
| Child distress after reopening |  |  | |  | |  | |  |  |  |  | |  | |  | |  |  | 0.15 | | 0.04 | | .18 | | 4.21** | |
| *R^2^* | .05** |  | |  | |  | |  |  | .47** |  | |  | |  | |  |  | .49** |  | |  | |  | |  |
| *F*(df_1_, df_2_) | *F*(4, 456) = 6.42** | | | | | | | |  | *F*(14, 446) = 28.31** | | | | | | | |  | *F*(15, 445) = 28.59** | | | | | | | |

*Note. B* = regression coefficient, *SE* = standard error, *β* = beta coefficient or standardized regression coefficient, *t* = t-value; *R^2^* = coefficient of determination;

*F*(df_1_, df_2_) = F-value and degrees of freedom.

* *p* < .05, ** *p* < .01, ^a^ 0 = no, 1 = yes, ^b^ 1 = one-parent family, 2 = two-parent family, ^c^ 0 = male, 1 = female

**Appendix S3**

**Childcare in Times of COVID-19 or CiToC questionnaire**

The following questions concern your and your child's experiences during the closure of the child care centers and after they reopened.

1. How did you experience the closure of the child care center?

| 1 = strongly disagree, 2 = disagree, 3 = somewhat agree, 4 = agree, 5 = strongly agree,  n.a. = not applicable | | |
| --- | --- | --- |
| 1. I agreed with the decision to close the child care centers.* | 1 2 3 4 5 | n.a. |
| 1. I found it easy to find alternative child care during the closure. | 1 2 3 4 5 | n.a. |
| 1. I enjoyed spending more time with my child during the closure. | 1 2 3 4 5 | n.a. |
| 1. I found it difficult to entertain my child during the closure. | 1 2 3 4 5 | n.a. |
| 1. I found it challenging to be a good parent during the closure. | 1 2 3 4 5 | n.a. |
| 1. I found it supportive that the child care center contacted me during the closure.* | 1 2 3 4 5 | n.a. |
| 1. I found it stressful to perform my caring responsibilities properly during the closure. | 1 2 3 4 5 | n.a. |
| 1. I found it stressful to combine my caring responsibilities with my work during the closure. | 1 2 3 4 5 | n.a. |

2. How did your child experience the closure of the child care center?

| 1 = strongly disagree, 2 = disagree, 3 = somewhat agree, 4 = agree, 5 = strongly agree,  n.a. = not applicable | | |
| --- | --- | --- |
| 1. My child struggled with the breakdown of the normal routine. | 1 2 3 4 5 | n.a. |
| 1. My child enjoyed their time at the alternative child care arrangement.* | 1 2 3 4 5 | n.a. |
| 1. My child enjoyed spending more time with me during the closure.* | 1 2 3 4 5 | n.a. |
| 1. My child found it difficult to entertain themselves during the closure. | 1 2 3 4 5 | n.a. |
| 1. My child missed the contact with the professional caregivers. | 1 2 3 4 5 | n.a. |
| 1. My child missed the contact with the other children at the child care center. | 1 2 3 4 5 | n.a. |
| 1. My child missed the play facilities, activities, and challenges offered at the child care center. | 1 2 3 4 5 | n.a. |

3. In the first two weeks after reopening, how did you feel about your child returning to child care?

| 1 = strongly disagree, 2 = disagree, 3 = somewhat agree, 4 = agree, 5 = strongly agree | |
| --- | --- |
| 1. I was a bit nervous about bringing my child to the child care center again. | 1 2 3 4 5 |
| 1. I liked being able to bring my child to the child care center again. | 1 2 3 4 5 |
| 1. I didn't have a problem with bringing my child to child care again. | 1 2 3 4 5 |
| 1. I was sorry to bring my child to the child care center again. | 1 2 3 4 5 |
| 1. I found it difficult to bring my child to the child care center again. | 1 2 3 4 5 |
| 1. I would have preferred to keep my child home. | 1 2 3 4 5 |
| 1. I felt confident that it was okay to bring my child to the child care center again. | 1 2 3 4 5 |
| 1. I was afraid that my child would contract the coronavirus and become sick. | 1 2 3 4 5 |
| 1. I was afraid that my child would contract the coronavirus and that I would become sick. | 1 2 3 4 5 |
| 1. I was afraid that my child would contract the coronavirus and that someone else would become sick. | 1 2 3 4 5 |

4. How did your child react when you dropped them off and collected them from child care in the first two weeks after reopening? You can of course discuss this with your partner if necessary.

| 1 = never, 2 = rarely, 3 = sometimes, 4 = often, 5 =always | |
| --- | --- |
| 1. My child was happy when dropped off at the child care center. | 1 2 3 4 5 |
| 1. My child was angry when dropped off at the child care center. | 1 2 3 4 5 |
| 1. My child cried when dropped off at the child care center. | 1 2 3 4 5 |
| 1. My child was anxious when dropped off at the child care center. | 1 2 3 4 5 |
| 1. My child was unsettled when dropped off at the child care center. | 1 2 3 4 5 |
| 1. My child didn't seem to mind when I left after dropping them off at the child care center. | 1 2 3 4 5 |
| 1. My child was happy when collected from the child care center.* | 1 2 3 4 5 |
| 1. My child was angry when collected from the child care center.* | 1 2 3 4 5 |
| 1. My child cried when collected from the child care center.* | 1 2 3 4 5 |
| 1. My child was anxious when collected from the child care center.* | 1 2 3 4 5 |
| 1. My child was unsettled when collected from the child care center.* | 1 2 3 4 5 |
| 1. My child seemed relieved when collected from the child care center. | 1 2 3 4 5 |

5. How did your child react when they saw the professional caregivers? This is about the first drop-off after the child care reopened. You can of course discuss this with your partner if necessary.

| 1 = strongly disagree, 2 = disagree, 3 = somewhat agree, 4 = agree, 5 = strongly agree | |
| --- | --- |
| 1. My child seemed to recognize the professional caregivers. | 1 2 3 4 5 |
| 1. My child immediately seemed to feel at ease with the professional caregivers. | 1 2 3 4 5 |
| 1. My child seemed indifferent to seeing the professional caregivers.* | 1 2 3 4 5 |
| 1. My child smiled at the professional caregivers. | 1 2 3 4 5 |
| 1. My child did not like being touched or picked up by the professional caregivers. | 1 2 3 4 5 |
| 1. My child tried to avoid contact with the professional caregivers. | 1 2 3 4 5 |
| 1. My child immediately went up to the professional caregivers. | 1 2 3 4 5 |

* = these items were not included in the current paper, based on the non-linear PCA

Scoring of the CiToC questionnaire:

Reverse the following items so that 1 = 5, 2 = 4, 3 = 4, 4 = 2 and 5 = 1: 1, 2, 3, 6, 10, 11, 17, 18, 22, 26, 31, 32, 38, 39, 41, 44

Calculate the mean of the following items to get the subscale Child Distress after Reopening: 26 - 44

Calculate the mean of the following items to get the subscale Parental Distress after Reopening: 16 - 22

Calculate the mean of the following items to get the subscale Child Stress during Closure:

9 - 15

Calculate the mean of the following items to get the subscale Parental Stress during Closure:

1 - 8

Calculate the mean of the following items to get the subscale Parental Fear of COVID-19:

23 - 25
